# MOB-suite: software tools for clustering, reconstruction and typing of plasmids from draft assemblies

**DOI:** 10.1099/mgen.0.000206

**Published:** 2018-07-27

**Authors:** James Robertson, John H. E. Nash

**Affiliations:** ^1^​ National Microbiology Laboratory, Public Health Agency of Canada, 110 Stone Road West, Guelph, ON, N1H7Y3, Canada; ^2^​ National Microbiology Laboratory, Public Health Agency of Canada, 180 Queen Street West, 11th Floor, Toronto, ON, M5V 1Z4, Canada

**Keywords:** plasmids, replicon benchmarking, mobile genetic elements, bacterial genomes, relaxase typing, plasmid transmissibility

## Abstract

Large-scale bacterial population genetics studies are now routine due to cost-effective Illumina short-read sequencing. However, analysing plasmid content remains difficult due to incomplete assembly of plasmids. Bacterial isolates can contain any number of plasmids and assembly remains complicated due to the presence of repetitive elements. Numerous tools have been developed to analyse plasmids but the performance and functionality of the tools are variable. The MOB-suite was developed as a set of modular tools for reconstruction and typing of plasmids from draft assembly data to facilitate characterization of plasmids. Using a set of closed genomes with publicly available Illumina data, the MOB-suite identified contigs of plasmid origin with both high sensitivity and specificity (95 and 88 %, respectively). In comparison, plasmidfinder demonstrated high specificity (99 %) but limited sensitivity (50 %). Using the same dataset of 377 known plasmids, MOB-recon accurately reconstructed 207 plasmids so that they were assigned to a single grouping without other plasmid or chromosomal sequences, whereas plasmidSPAdes was only able to accurately reconstruct 102 plasmids. In general, plasmidSPAdes has a tendency to merge different plasmids together, with 208 plasmids undergoing merge events. The MOB-suite reduces the number of errors but produces more hybrid plasmids, with 84 plasmids undergoing both splits and merges. The MOB-suite also provides replicon typing similar to plasmidfinder but with the inclusion of relaxase typing and prediction of conjugation potential. The MOB-suite is written in Python 3 and is available from https://github.com/phac-nml/mob-suite.

## Data Summary

1. Supplementary methods, tables and figures have been deposited in Figshare; DOI: 10.6084/m9.figshare.6177188 (https://figshare.com/s/19be2e8de76cf43eab44).

**Impact Statement**

Plasmids facilitate the rapid adaptation of bacteria and are of increasing concern due to the presence of antimicrobial resistance (AMR) determinants on plasmids. Whole genome sequencing is becoming routine for many foodborne bacterial pathogens, which has greatly increased knowledge of their genetic diversity. Analyses of plasmid content are complicated because sequencing technologies currently employed do not provide complete chromosomes or plasmid sequences. Disentangling the plasmid content from the chromosome allows the tracking of plasmids through populations and understanding the spread of traits such as AMR. The MOB-suite was designed as a set of modular tools for facilitating the reconstruction and typing of plasmids from draft assemblies and provides a significant improvement over several tools commonly cited in the literature.

## Introduction

Whole genome sequencing (WGS) using short reads is becoming routine for microbial genomes as laboratories switch from traditional phenotypic diagnostics. The amount of raw WGS data available for pathogens such as *Salmonella* and *Escherichia coli* has increased greatly with more than 200 000 samples deposited in the NCBI short read archive (SRA). However, assemblies generated using Illumina sequencing do not produce complete genomes, which has frustrated efforts to characterize the plasmid content of samples [1–4]. In part this is because Illumina technology produces short reads while plasmids tend to contain repeat sequences with sizes greater than sequences generated by Illumina technology. As of May 2018, there are 12 091 complete bacterial plasmids and 9461 complete chromosome sequences in NCBI’s Refseq. High-throughput purification of plasmid DNA is intractably difficult for large plasmids (>100 kb), so numerous methods have been developed to extract plasmid from chromosomal sequences using *in silico* tools, with plasmidfinder [4] being the most extensively cited.

Plasmids can rapidly disseminate antimicrobial resistance (AMR) traits through bacterial populations and so identifying and tracking them are critical to mitigation strategies for AMR [5, 6]. Conjugation is the most effective method for horizontal transmission of plasmids and involves numerous proteins, which mediate the transfer from the donor cell and establishment in the recipient cell [7–11]. A conjugative plasmid contains the complete set of genes and DNA features needed for transfer including an origin of transfer (oriT), a DNA relaxase, a type IV coupling protein (T4CP) and the type IV secretion system (T4SS) [7, 9, 10, 12]. A transmissible plasmid must possess at a minimum an oriT and usually a relaxase but this can be provided *in trans* [7, 8, 10]. Numerous typing schemes have been developed for plasmids [7, 11, 13] but replicon typing has served as the standard method for categorizing plasmids based on the DNA sequences responsible for replication of the plasmid [4, 7, 11, 14]. MOB typing is the other commonly used typing scheme, which is based on the N terminus of the relaxase protein from the transfer module of the plasmid [7, 11, 12]. Relaxase typing is limited in its utility because no tools are available to perform automatic *in silico* relaxase typing for query sequences. Relaxases are known to be important for identifying the host range for plasmids, and a large-scale analysis of the distribution of conjugative transfer system sequences has shown that the presence of some relaxase classes differs between divergent taxonomic groups, while other classes are widespread [15].

There is a strong need for automatic tools for the detection of plasmids from WGS data and numerous tools have been developed using a diverse array of approaches [1, 4, 16–18]. Assembly-based approaches using short reads will result in fragmented plasmids and chromosomes and the assembly process has been shown to possess a decreased sensitivity for the detection of some resistance genes, compared to methods using the raw reads, due to variable coverage of target regions and choice of assembly algorithm [19]. Plasmidfinder utilizes a database of marker sequences associated with replicon types of plasmids and is available as a web-based tool [4]. cBAR identifies plasmid sequences based on 5-mer compositional differences [20]. The plasmidSPAdes tool exploits differences in coverage of chromosomal and plasmid sequences to extract them from the de Brujin graph [21]. A drawback to the coverage dependency of plasmidSPAdes is that large and low-copy plasmids will be nearly indistinguishable from the chromosome [18], which coverage-agnostic approaches such as plasmidfinder, cBAR and MOB-suite do not have. In previous benchmarks, it was shown that successful plasmid detection with plasmidSPAdes requires at least 40× coverage and differential chromosome coverage compared to the plasmid [18]. PlasmidTron uses a genome-wide association (GWAS) approach for reconstruction of plasmids responsible for specific phenotypes such as AMR [18], but we limited our benchmarking to tools that do not require any *a priori* knowledge for plasmid identification. A full discussion of all of the approaches is beyond the scope of this paper and a summary of the existing tools is provided by Orlek *et al*. [11]. Benchmarking of multiple methods has been the subject of two recent papers and we selected the best performing tools which are commonly used for our comparisons [1, 16].

Here we present the MOB-suite of tools for the typing and reconstruction of plasmid sequences from WGS assemblies (https://github.com/phac-nml/mob-suite) and benchmark it against three popular tools. The MOB-suite is a modular set of tools for the clustering, reconstruction and typing of plasmids from assemblies. It uses a reference database approach for identifying contigs of plasmid origin and then aggregates the plasmid contigs into groups based on an internal clustering scheme. As input, the MOB-suite accepts fasta-formatted genome assemblies produced by any assembler. Additionally, the suite provides a scalable nomenclature for identifying plasmids over short evolutionary periods by estimating genomic distances based on mash min-hashing [22]. The output of each of the tools can be incorporated into pipelines depending on the application of the end-user.

## Theory and implementation

The MOB-suite encompasses three linked modules for analysis: MOB-cluster, MOB-recon and MOB-typer.

### MOB-cluster method

MOB-cluster uses mash dist [22] with default parameters to calculate all pairwise genomic distances for each plasmid contained in the closed plasmid reference database. Details on the construction of this dataset are described in the Supplementary Methods and the host taxonomic composition of the plasmids is presented in Fig. S1 using KRONA [23]. Single-linkage clustering is performed using the fcluster [24] package from SciPy at two default distance thresholds (0.05, 0.001), although the software permits user-defined thresholds. The default thresholds are heavily optimized for publicly available *Enterobacteriaceae* plasmids and these may not be appropriate for other taxa of interest. The cluster code information is then incorporated into a fasta file header ready to be used by MOB-recon and MOB-typer. These cluster codes are used to broadly group reference plasmids with similar sequence content together for the purposes of comparing plasmid similarity and aggregating contig sequences to similar plasmid backbones. However, a single genetic distance threshold for delimiting meaningful sequence clusters will not accurately partition all of the plasmid variability. We selected a permissive mash dist clustering threshold of 0.05, which resulted in clusters consisting largely of single replicon and MOB types. The distance metric used by mash dist allows for plasmids with considerable differences in size to be grouped together. This is desirable for the purposes of plasmid reconstruction, largely because plasmids can undergo large changes in their sequence content. A given contig sequence may share identical sequence identity with a diverse array of plasmids and the permissive clustering level is used to prioritize which cluster code the contig should be assigned to in the winner-take-all strategy of MOB-recon. The clustering process can incrementally update the database with one or more records as well as build an entirely new database. The clustering algorithm will only assign a new cluster if the new sequences fall outside the defined sequence thresholds for the database, which allows users to use the same cluster designations for their reference sequences even after updating their database. Only high-quality and closed reference plasmids should be incorporated into the database because the other tools depend on the plasmid sequences and cluster information. Low-quality sequences or chromosomal contaminants will degrade the performance of the tools and can result in the creation of spurious clusters.

### MOB-recon method

**Fig. 1.**
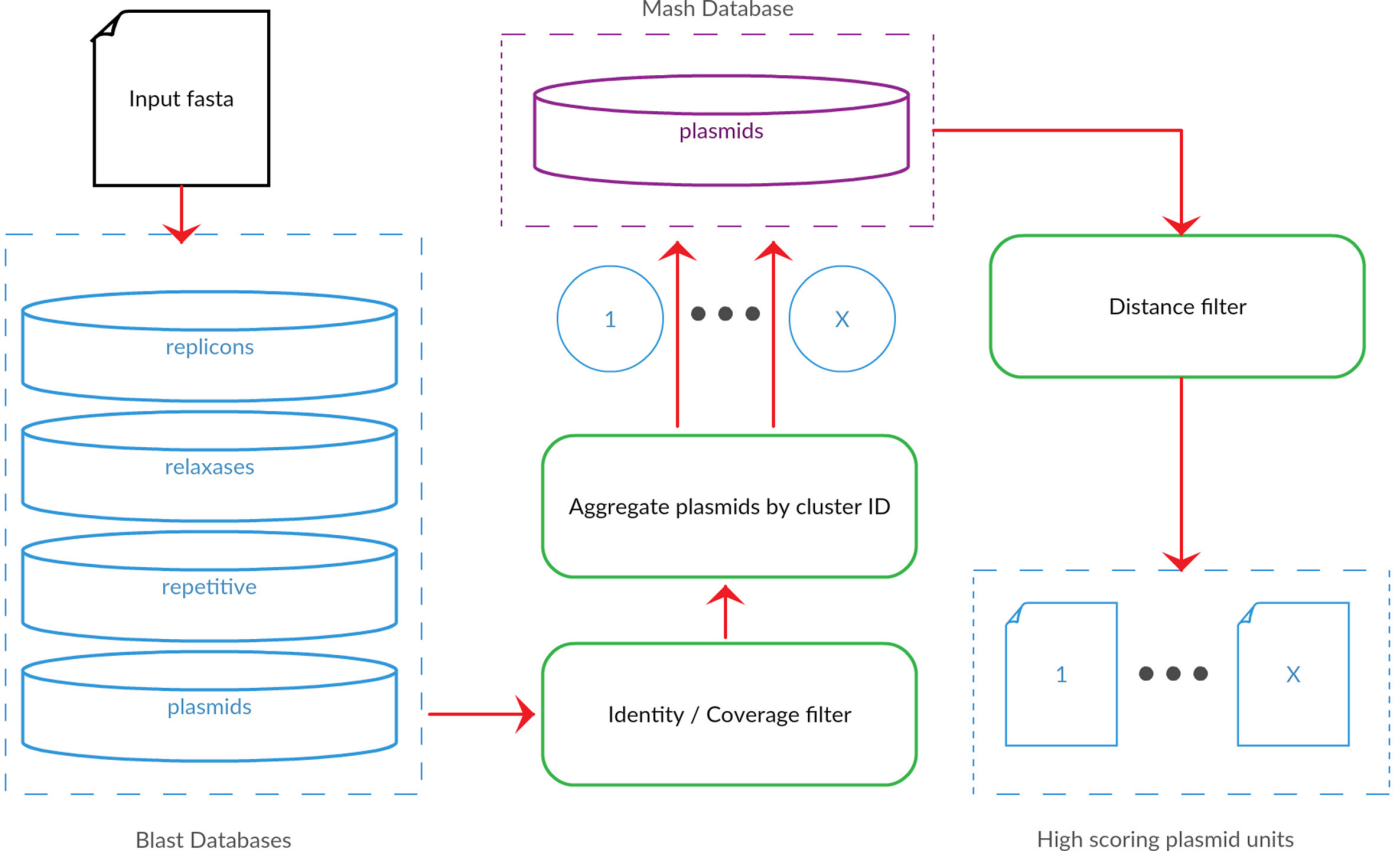
Flowchart outlining the major elements of the MOB-recon algorithm. Draft or complete assemblies are used as input to the software and candidate contigs of plasmid origin are identified and clustered together to produce a report file and individual fasta files for each grouping.

MOB-recon is an ensemble-based approach using marker sequence databases of known replicons and relaxases along with a curated database of complete plasmids clustered together using MOB-cluster; an overview of the algorithm is described in Fig. 1. The user supplies a draft or complete assembly using the assembler of their choice, although unicycler [3] is recommended due to the automatic circularization and pilon [25] error correction within the pipeline, and the algorithm can accept input from any assembly pipeline. MOB-recon will check the fasta headers for circularity status for the presence of the ‘circular=true’ flag. If contigs have been circularized using other assemblers, the contigs can be identified as circular by adding that flag to the header. Optionally, the minimus2 tool provided by Circlator [26] is run on the assembly to determine if any contigs have overlapping ends. All circular contigs are considered putative plasmids and will be included in the final results regardless of the other filters applied. Known replicons and relaxases are used as queries to blast against the input assembly and the results are filtered for coverage and identity with the overlapping hits removed by selecting the hit with the highest bit score. Contigs with a relaxase or a replicon are considered as candidate contigs. A set of repetitive DNA elements were retrieved from NCBI ENTREZ using the query ‘mobile_elements[FEAT] AND bacteria[organism]’ and the repetitive elements were parsed from the flat files to construct a database of known repetitive elements. The repetitive elements database is used to flag problematic contigs, which are associated with plasmids but are not sufficient to identify the presence of a plasmid. This prevents the prediction of plasmids, which consist of nothing but repetitive elements. The classified insertion sequence (IS) elements were parsed from the GenBank flat file and single queries were made to the ISFinder database [27] to classify IS elements not assigned to an IS family. Finally, the assembly is used as a query to a database of complete plasmids and hits are filtered based on user-supplied coverage and identity.

Contig sequences are aggregated into putative plasmid units according to the reference hits obtained from the complete plasmid reference database using the cluster code information contained in the fasta header. A contig can only be assigned to one plasmid unit or to the chromosome, which means that repetitive elements with multiple copies will only be assigned to one unit. Each reference sequence is scored according to the cumulative non-overlapping bit score hits for each query sequence and a priority queue is established by ranking the reference plasmid clusters according to the highest blast bit score. Each query is then assigned to the highest-ranking reference plasmid cluster code to which the query had a hit. The initial plasmid units are then checked for circular sequences and if there are multiple sequences the circular sequence is split into its own unit. If a plasmid unit consists of nothing but repetitive DNA elements without a known replicon or relaxase, the cluster is discarded. The grouped sequences representing the plasmid unit are then written to individual fasta files and mash is used to identify the closest reference plasmid to the reconstructed plasmid. If the distance is greater than the cluster threshold (0.05) then the plasmid is labelled as a novel group. A fasta file of the chromosome sequences with the plasmid sequences removed is written along with a report for each contig sequence.

### MOB-typer method

**Fig. 2.**
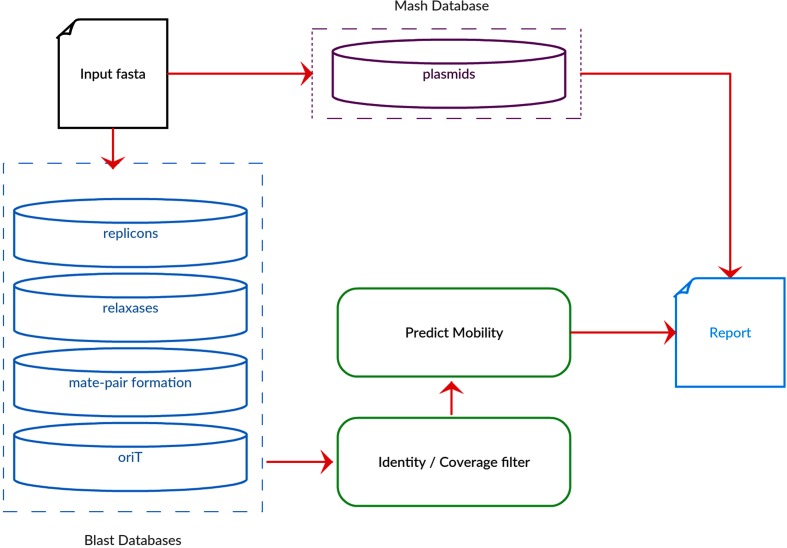
Flowchart outlining the major elements of the MOB-typer algorithm. Draft or complete assemblies are used as input to the software and each plasmid is typed using known replicons and relaxases. Additional databases of mate-pair formation proteins and known oriT sequences are used to predict the transmissibility of the plasmid.

MOB-typer provides replicon typing similar to plasmidfinder but with the inclusion of relaxase typing, oriT predictions and conjugative transferability predictions. An expanded set of plasmid-derived replication proteins was assembled using the initial set of probes derived from plasmidfinder but also including known replication proteins not associated with a known incompatibility group. Relaxase and mate-pair formation databases were constructed using the original Shintani *et al.* queries from their table S2 [7] and expanded to a comprehensive set of queries (Supplemental Methods). Details on the expansion of each marker sequence dataset are described in the Supplemental Methods. Known oriT sequences were extracted from the NCBI GenBank annotations for the reference plasmids and were assigned to MOB types by matching them to their relaxase on the plasmid. An overview of the MOB-typer workflow is presented in Fig. 2. Each of the different marker databases are used as blast queries against each assembly file and the results are compiled into a report. The mobility prediction is based on the presence of relaxase, mate-pair formation and oriT sequences. A plasmid is classified as ‘conjugative’ if it contains at least a relaxase and a mate-pair formation marker. Plasmids containing either a relaxase or an oriT but are missing the mate-pair formation marker are classified as ‘mobilizable’, while plasmids that are missing a relaxase and an oriT are classified as ‘non-mobilizable’.

### Benchmarking plasmid detection

**Fig. 3.**
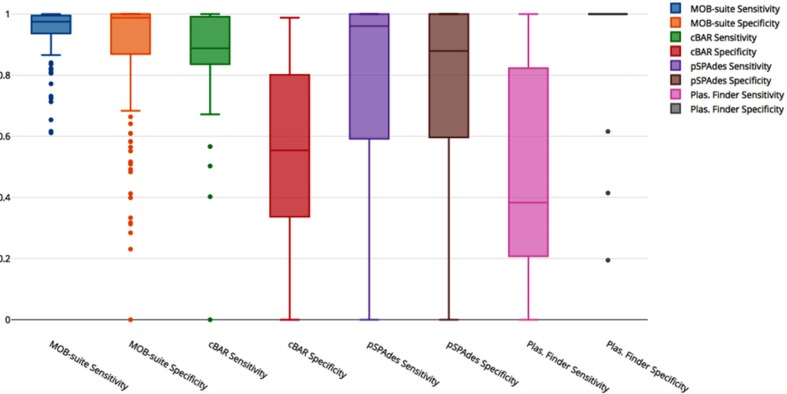
Box-plot outlining the sensitivity and specificity for each of the tested tools.

We selected 133 closed genomes with 377 associated plasmids in GenBank, which were sequenced using PacBio and Illumina technologies (Table S1). Cluster information for all of the reference genomes is available in Table S2. We compared the performance of MOB-recon with three other commonly used plasmid recovery tools: cBAR, plasmidSPAdes and plasmidfinder, and a full description of the methods used is presented in the Supplementary Methods. The Illumina data were assembled using unicycler v.0.4.3 with default parameters [3]. The resulting assemblies were used as blastn v.2.6.0 [28] queries against their respective closed genome assembles with the following options (-max_hsps 1 -num_alignments 1 -perc_identity 50 -qcov_hsp_perc 50). Any contigs not found in the closed assembly with at least 50 % identity and coverage were discarded from further analyses. To determine the coverage of the closed references by the Illumina assembly the total closed reference bases covered by the assembly were summed. It is possible that the Illumina assembly could have multiple contigs representing the same sequence, and as a result, the number of bases mapping to the closed assembly can exceed the size of the closed assembly. It is known that certain sequences pose issues for Illumina sequencing [29] and when comparing the draft genomes against the final assembly deposited in NCBI, we found that Illumina-only assemblies covered on average 93 % of the closed assembly (Table S1). Sensitivity and specificity for each tool was determined as the number of bases correctly assigned to plasmid or chromosome of the Illumina-only assembly. The numbers of bases assigned to each category are presented in Table S1 and the sensitivity and specificity for each tool are presented in Fig. 3. MOB-recon demonstrates both high sensitivity and specificity at 95 and 88 %, respectively, while plasmidfinder demonstrated high specificity (99 %) but limited sensitivity (50 %). Both MOB-recon and plasmidfinder use replicon databases to detect plasmid contigs but MOB-recon benefits from an ensemble database approach which provides more sensitivity over the single replicon-based approach. cBAR had high sensitivity (88 %) but low specificity (55 %) while plasmidSPAdes showed more consistent performance with 78 and 77 % sensitivity and specificity, respectively.

### Benchmarking plasmid reconstruction

MOB-recon and plasmidSPAdes are the only tools which attempt to reconstruct the specific plasmid content from assemblies, and so the accuracies of these two tools were compared against the ground truth of their closed assemblies (Table 1). Each contig was assigned to the reference assembly based on blastn as described above. A total of 377 closed reference plasmids were included in this comparison, and 324 and 337 plasmids were identified by plasmidSPAdes and MOB-recon, respectively. Using the known reference assembly membership of each contig, we examined the proposed plasmid clusters as to how well they recapture the true sequence relationships. Correctly reconstructed plasmids required all of the contig sequences from the draft assembly to be present in a single group and within that group there were no contigs, which belong to either the chromosome or other plasmids. A split event is when the tool distributed the contigs of a plasmid into multiple plasmid units. A merge event is recorded when multiple plasmids are contained within a single plasmid unit. A hybrid event is when a plasmid has undergone both split and merge events. In general, plasmidSPAdes has a tendency to merge plasmids together, with 208 plasmids undergoing merge events, but it was able to accurately reconstruct 102 of the 324 identified plasmids. MOB-recon was able to accurately reconstruct 207 of the 337 plasmids it identified but it produced more mosaic plasmid units, with 84 plasmids undergoing both splits and merges.

**Table 1.** Performance benchmarking of the ability to reconstruct plasmid groups by plasmidSPAdes and MOB-recon Contigs were assigned to their respective plasmid by blast and the content of the groupings was evaluated. A plasmid is considered to have undergone a split event if contigs are present in more than one cluster. A merge event is defined as a cluster that contains multiple plasmids. Combination events are when a plasmid has undergone both a split and a merge. Correctly partitioned plasmids are ones that have been assigned to a single grouping and where no additional plasmid contigs have been assigned to that cluster.

	PlasmidSPAdes	MOB-recon
Total plasmids identified	337	377
Correctly partitioned plasmids	102	207
Plasmids split across multiple clusters	14	50
Plasmids merged into single clusters	208	36
Plasmids with a combination of splits and merges	13	84

## Conclusion

There is a great deal of interest in identifying plasmids from WGS draft assemblies, but extraction of plasmid sequences remains a difficult but not impossible task. The MOB-suite of tools provides a significant improvement in the identification of plasmid sequences from WGS assemblies. MOB-recon is also able to provide information regarding completeness of the plasmid, which none of the currently available tools provides. The MOB-suite provides novel functionality, which will be of use to researchers interested in plasmids. The cluster codes provided by MOB-cluster provide a mechanism for description of plasmids that share significant sequences without the need for defined biomarkers. These cluster codes provide internal consistency within a single database instance, allowing for comparisons between samples across multiple independent runs. However, the use of codes outside of a local user’s database is limited due to the need for centralization of cluster assignment, which is analogous to the centralized allele and sequence type assignments for multilocus sequence typing approaches [30–33]. MOB-typer is currently the only automatic typing tool for relaxase typing of plasmids and is the only tool to provide plasmid transmissibility predictions. This information is critical for evaluating the risk a plasmid poses for disseminating traits such as AMR through a population.

The MOB-suite depends on a comprehensive database of plasmids to provide highly accurate results and it will not perform well on novel plasmids that do not share significant sequence similarity to those in the database. The availability of plasmids in NCBI is skewed towards those from *Enterobacteriaceae* and work is needed to improve the coverage of plasmids from other taxonomic groups (Fig. S1). The aim of the MOB-suite is to improve the tools available for plasmid characterization, but long read sequencing using PacBio or Nanopore should be performed when plasmids fail to assemble fully using Illumina data only and the complete sequence of the plasmid is needed.

## Data bibliography

1. Robertson J, Nash JHE. FigShare. DOI: 10.6084/m9.figshare.6177188

## Supplementary Data

Supplementary File 1

## Supplementary Data

Supplementary File 2

## Supplementary Data

Supplementary File 3
