# Supplementary material for: MOB-suite: software tools for clustering, reconstruction and typing of plasmids from draft assemblies
Source: Microb Genom. 2018 Jul 27;4(8):e000206. doi: 10.1099/mgen.0.000206 (PMC6159552; doi:10.1099/mgen.0.000206)

Supplemental Table 1 – Performance metrics for each assembled isolate used in the benchmarking analysis of the MOB-suite

Supplemental Table 2 –MOB-typer report for all of the reference plasmids contained in the MOB-suite reference database

Supplemental Methods – Full methodological descriptions of the assembly of the marker sequence databases and benchmarking of the MOB-suite.

Supplemental Methods

Retrieving complete plasmid sequences and metadata from NCBI

The NCBI Entrez nucleotide database was queried on May 2017 with the query 'plasmid AND "complete sequence" AND bacteria[organism]'. The results were then filtered for sequence length between 1,500bp to 400,000bp and 'plasmid' as the genetic compartment. This yielded 20,111 initial sequences. The initial target list contained both Refseq and GenBank. The accession list was then filtered to remove any record from draft assemblies or which contained the words "CDS, gene, transposon, insertion sequence, incomplete". To remove duplicate sequences, Refseq accessions, which contained the accession of another record were removed which resulted in 12,091 plasmids recovered.

Building replicon, relaxase, MPF, oriT databases

The Plasmid Finder database was downloaded on May 2017 from (http://www.genomicepidemiology.org/) and used locally. Initial relaxase and MPF queries were downloaded from the supplemental material from Shintani et. al 2015. NCBI complete plasmids were annotated using Prokka v. 1.19 (https://github.com/tseemann/prokka). Replicon, relaxase and MPF sequences were matched against the database of gene sequences using blastn with a threshold of 80% identity and coverage. After the initial round of blastn, the protein sequences for the matched queries was then used to query the database again, to capture distant hits with manual curation of the hits based on a minimum e-value of 1e-10 as well as the annotations of the matched sequences. This process was completed iteratively until no new acceptable matches were found. Matches for replicons, relaxases and MPF proteins were clustered using CD-HIT v. 4.7 (http://weizhongli-lab.org/cd-hit/) using a range of thresholds (0.70,0.80,0.90,0.95). Singleton clusters were reviewed and blasted against the NCBI public protein database and curated to remove incorrect records.

Assignment to replicon, MOB and MPF categories was performed by majority voting in clusters according to the initial seed queries and in the case of novel proteins given an id based on the founding member of the protein cluster. In the case of MOB and MPF types, this process was guided by the assignments given in the supplemental material of Shintani et. al 2015 for the plasmids which were included in their work. To account for the RNA determinants of replicon type the initial replicon markers from Plasmid Finder, which could not be assigned to a gene were added into the gene based DNA sequences to make up the replicon marker database. NCBI plasmid annotation with oriT features were extracted from the database of

plasmids built previously and augmented with literature searches for known oriT sequences

Genome Assembly

Raw Illumina reads were downloaded from the SRA for each of the samples listed in the Supplemental Table 1. These were assembled using unicycler v. 0.4.3 (https://github.com/rrwick/Unicycler) with the default mode and only Illumina reads. The resulting assemblies were used as blastn v. 2.6.0 (https://blast.ncbi.nlm.nih.gov/Blast.cgi?PAGE_TYPE=BlastDocs&DOC_TYPE=Download) queries against their respective closed genome assembles with the following options (-max_hsps 1 -num_alignments 1 -perc_identity 50 -qcov_hsp_perc 50). Any contig not found in the closed assembly with at least 50% identity and coverage were discarded from further analysis. The bases mapping to each closed sequence in the assembly was summed to determine the coverage of the close references in the Illumina assembly. It is possible that the Illumina assembly could have multiple contigs representing the same sequence and because of this, the number of bases mapping to the closed assembly can exceed the size of the closed assembly.

Plasmid detection

Unicycler assemblies were used as input to cBAR v. 1.2 (http://csbl.bmb.uga.edu/~ffzhou/cBar/) and MOB-recon v. 1.0 (https://github.com/jrober84/mob-suite). The plasmid finder (https://cge.cbs.dtu.dk/services/PlasmidFinder/) database was downloaded in November 2017 and used as blastn queries against the unicycler assemblies with the following options (-evalue 1e-10 -perc_identity 95 -qcov_hsp_perc 60). Best blast hits were selected from the results using the blast_best_hits.py provided by the MOB-suite. Plasmid spades v. 3.9.0 (http://spades.bioinf.spbau.ru/) is the only tool which used the raw Illumina data as input and was run with the default options. Plasmid contigs identified by plasmid SPAdes were blasted against their closed assemblies in the same way as the unicycler assemblies.

Supplemental Figure 1 – KRONA plot of the taxonomic composition of the closed plasmid reference database used by the MOB-suite

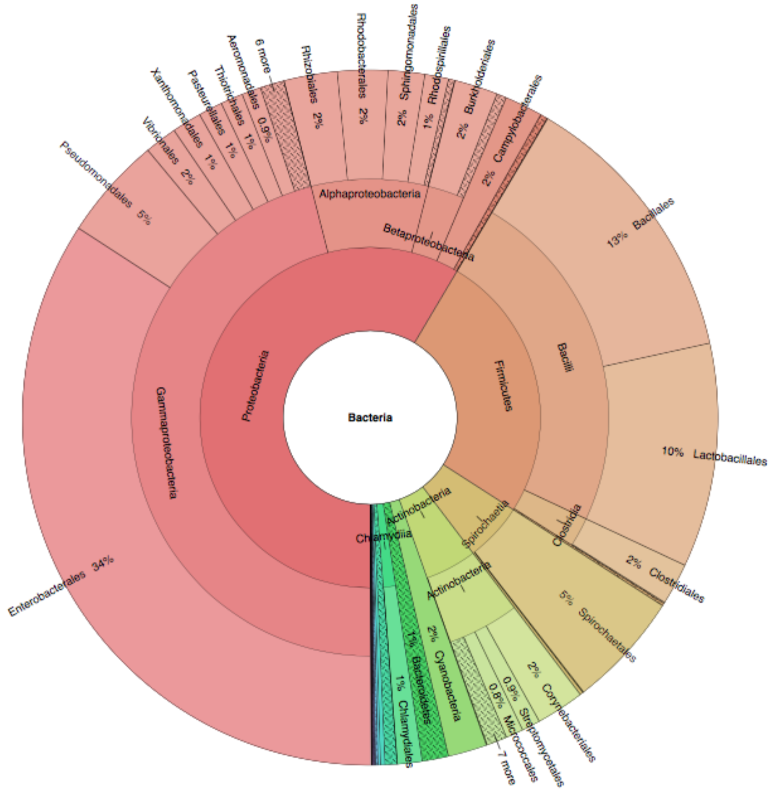

Supplement: Supplementary File 1 [file mgen-5-206-s001.pdf]
